# Early Outcomes in Severely Obese Patients Undergoing Sternum-Sparing Minimally Invasive Multivessel Artery Bypass Grafting Using Total Coronary Revascularization via Left Anterior Mini-Thoracotomy

**DOI:** 10.3390/jcm14082545

**Published:** 2025-04-08

**Authors:** Volodymyr Demianenko, Markus Schlömicher, Marius Grossmann, Ahmed Belmenai, Hilmar Dörge, Christian Sellin

**Affiliations:** Department of Cardiothoracic Surgery, Heart-Thorax Center, Klinikum Fulda, University Medicine Marburg, Campus Fulda, Pacelliallee 4, 36043 Fulda, Germany; markus.schloemicher@klinikum-fulda.de (M.S.); marius.grossmann@klinikum-fulda.de (M.G.); ahmed.belmenai@klinikum-fulda.de (A.B.); doerge@klinikum-fulda.de (H.D.); christian.sellin@klinikum-fulda.de (C.S.)

**Keywords:** obesity, surgical outcomes, minimally invasive cardiac surgery, CABG, TCRAT

## Abstract

**Background/Objectives:** Severe obesity significantly increases the risk of complications following full sternotomy in coronary artery bypass grafting (CABG). However, these patients are frequently excluded from less invasive, sternum-sparing surgical alternatives. This study aimed to assess the safety and practicality of a newly developed technique—Total Coronary Revascularization via left Anterior miniThoracotomy (TCRAT)—that avoids sternotomy in patients with severe obesity requiring multivessel CABG. **Methods***:* From November 2019 to May 2024, a total of 502 non-emergency patients with multivessel coronary artery disease underwent CABG through a left anterior minithoracotomy using cardiopulmonary bypass (CPB) and cardioplegic arrest. Of these, 43 patients with a body mass index (BMI) exceeding 35.0 kg/m^2^ were classified as severely obese and included for subgroup analysis. Their outcomes were compared to those of the remaining 459 patients with BMI below 35.0 kg/m^2^. Key intraoperative variables—such as total operative time, CPB duration, aortic cross-clamp time, and graft strategy—were evaluated. Postoperative outcomes, such as the incidence of major adverse cardiac and cerebrovascular events, minor complications, and length of stay in ICU and hospital, were also analyzed. **Results**: Severely obese patients exhibited a longer total operation time (353.5 ± 83.6 min vs. 320.4 ± 73.4 min, *p* < 0.05). In contrast, no statistical differences were observed in aortic cross-clamp time (97.9 ± 27.6 min vs. 95.6 ± 33.0 min; *p* = 0.307) or CPB time (163.3 ± 35.0 min vs. 155.0 ± 42.9 min; *p* = 0.078). Both groups received a similar number of distal anastomoses (3.1 ± 0.7 vs. 3.0 ± 0.8; *p* = 0.194), and the frequency of total arterial revascularization was comparable (34.9% vs. 40.0%; *p* = 0.268). There were no differences between the groups in major complications, including hospital mortality (2.3% vs. 1.1%, *p* = 0.227), stroke (0.0% vs. 0.6% *p* = 0.300), or need for re-revascularization (0.0% vs. 1.1%, *p* = 0.248). Similarly, minor complications, such as wound healing issues (2.3% vs. 1.1%, *p* = 0.233) and revisions for bleeding (4.6% vs. 7.2%, *p* = 0.276), were comparable between groups. ICU stay (2.7 ± 4.5 days vs. 2.2 ± 4.0 days; *p* = 0.225) and total hospital stay (12.3 ± 9.6 days vs. 10.8 ± 8.6 days; *p* = 0.142) showed no meaningful differences. **Conclusions**: TCRAT can be performed safely and effectively in severely obese patients, providing a feasible minimally invasive option for complete coronary revascularization in cases of multivessel disease. This approach eliminates the complications associated with sternotomy, making it a valuable surgical alternative for this high-risk patient group.

## 1. Introduction

Obesity is a significant global health issue, contributing to numerous comorbidities such as cardiovascular disease, diabetes, and hypertension [1]. The rising prevalence of obesity poses challenges for surgical procedures, particularly those involving the cardiovascular system [2].

Total Coronary Revascularization via left Anterior Thoracotomy (TCRAT) emerged as a minimally invasive surgical approach in the treatment of coronary artery disease [3,4,5]. This procedure is routinely applicable to a broad range of patients and demonstrates promising early and midterm outcomes [4,6]. It utilizes established surgical techniques, including cardiopulmonary bypass (CPB), aortic cross-clamping, cardioplegic cardiac arrest, and standard anastomotic techniques. Regarding the applicability and perioperative outcome of the TCRAT technique even in severely obese patients, the data available are very limited.

Previous research has documented the challenges obesity presents in various surgical contexts, notably in cardiovascular procedures. Various studies have shown that obesity can lead to increased operative time, higher rates of wound site infections, and extended hospital stays. It has been indicated that obese patients undergoing coronary artery bypass grafting (CABG) have higher incidences of wound infections and respiratory complications. Similarly, it has been reported that obese patients experience longer operative times and a higher risk of postoperative complications in cardiac procedures [7,8].

Conversely, some studies suggest that obese patients can have outcomes comparable to non-obese patients [9,10]. Research on minimally invasive cardiac procedures has highlighted that obese patients can achieve similar success rates and complication profiles to their non-obese counterparts when meticulous surgical techniques and perioperative care are employed. Although obese patients undergoing cardiac surgery had longer procedural times, their in-hospital outcomes did not significantly differ from those of non-obese patients. However, the burden of obesity manifests in substantially increased rates of wound healing disorders and sternal reconstructions.

Studies concerning the technique of minimally invasive off-pump coronary artery bypass grafting (MICS) indicate relative contraindications for this approach, particularly in patients with a large body habitus. The challenges of performing MICS in obese patients stem from technical difficulties and an increased risk of conversion to sternotomy. These contraindications necessitate careful patient selection and consideration of alternative surgical approaches to mitigate the risks associated with severe obesity [11,12].

The variability in these findings underlines the need for procedure-specific data. TCRAT offers potential benefits in reducing surgical trauma and recovery time. However, the specific impact of obesity on TCRAT outcomes is unclear. This study aims to evaluate the in-hospital outcomes following TCRAT in severely obese patients.

## 2. Materials and Methods

### 2.1. Study Design and Patient Population

The present study included 502 consecutive patients who underwent TCRAT from November 2019 to May 2024. All technical aspects of the surgical technique employed in this study have been thoroughly described and illustrated in detail by various groups of authors in previous publications [3,4,5,6], including supplementary video materials, thereby providing a well-documented foundation for reproducibility. A BMI of more than 35.0 kg/m^2^ was defined as severe obesity. The patient population was stratified into two distinct groups predicated on BMI: Group A included patients with a BMI less than 35.0 kg/m^2^, while Group B comprised those with a BMI exceeding 35.0 kg/m^2^.

All patients had multivessel coronary artery disease.

Patient selection for the TCRAT operation was the result of thorough deliberation by the heart team, following guideline indications to determine which coronary arteries necessitated grafting [13]. Complete anatomic revascularization was characterized by the successful treatment of all significant coronary lesions, defined as those with a visually estimated diameter stenosis of 50% or greater in vessels with a reference diameter of at least 1.5 mm [14].

Exclusion criteria for TCRAT comprised patients classified as emergency cases necessitating immediate catheterization and surgery, those presenting with significant atheromatous disease of the ascending aorta, moderate to severe aortic regurgitation, significant valvular heart disease, individuals undergoing repeat operations, and those with a history of prior CABG.

### 2.2. Data Collection

Comprehensive data were gathered from patient medical records, encompassing preoperative, intraoperative, and postoperative phases. Preoperative data included patient demographics, BMI, comorbid conditions such as diabetes and chronic obstructive pulmonary disease, and records of preoperative coronary interventions. Intraoperative data included total operation time, aortic cross-clamp time, CPB time, the number of distal anastomoses performed, and graft configuration.

The postoperative outcomes included the length of stay in the ICU, the incidence of delirium, atrial fibrillation, pneumonia, wound infections, the total hospital stay, the need for hemodialysis, the incidence of stroke, re-revascularization events, revisions for bleeding, and in-hospital mortality rates.

Postoperative myocardial infarction was stringently defined as an increase in CK-MB levels within 48 h post-procedure, reaching up to 10 times the local laboratory’s upper limit of normal (ULN), or five times the ULN accompanied by newly occurring Q-waves in two contiguous leads or a new persistent left bundle branch block, adhering to the Fourth Universal Definition of Myocardial Infarction by the Society for Cardiovascular Angiography and Interventions (SCAI) [15].

The definition of postoperative stroke aligned with the updated criteria from the American Heart Association and American Stroke Association for the 21st century. Stroke was delineated as a neurological deficit attributable to an acute focal injury of the central nervous system due to a vascular cause, encompassing cerebral infarction, intracerebral hemorrhage, and subarachnoid hemorrhage [16].

As part of the preoperative screening, all patients underwent CT-angiography in addition to the standard institutional examinations, aimed at identifying calcification, anatomical abnormalities and atherosclerotic disease in the ascending aorta, aortic arch, and major arterial branches, including the iliac and femoral vessels.

### 2.3. Statistical Analysis

Continuous variables were articulated as mean ± standard deviation (SD) and compared utilizing Student’s *t*-test. Statistical significance was determined with a *p*-value of less than 0.05. All statistical analyses were performed using SPSS software version 29.0.0.0 (IBM SPSS Statistics, Armonk, New York, NY, USA).

### 2.4. Ethical Standards

This study received approval from the local ethics committee (2021-2621-evBO) 28 December 2021 and was conducted in compliance with the ethical principles outlined in the Declaration of Helsinki of 1964 and its subsequent amendments.

### 2.5. Consent Statement

Written consent was obtained from all patients for the use of their data in scientific publications, ensuring adherence to ethical standards and patient confidentiality.

## 3. Results

### 3.1. Patient Characteristics

The average age of patients in Group A was 67.5 ± 9.7 years, with an average BMI of 27.5 ± 3.6 kg/m^2^. Patients in Group B were significantly younger, with an average age of 63.7 ± 7.3 years, and had an average BMI of 37.8 ± 2.2 kg/m^2^. Group B had a higher rate of diabetes, affecting 72.1% of patients, compared to only 32.9% in Group A. Despite differences in age, BMI, and diabetes prevalence, other preoperative characteristics remained notably comparable between the two groups. Gender distribution was evenly matched, and both groups exhibited comparable left ventricular ejection fraction and EuroSCORE II values. Baseline characteristics are given in Table 1.

### 3.2. Intraoperative Outcomes

Patients in Group B had a longer total operation time (353.5 ± 83.6 min) compared to Group A (320.4 ± 73.4 min). The aortic cross-clamp time and CPB time were similar between groups.

Additionally, the number of distal anastomoses was similar between the groups, with Group A averaging 3.0 ± 0.8 (range 2–5) and Group B averaging 3.1 ± 0.7 (range 2–4). Operative data are given in Table 2.

### 3.3. In-Hospital Outcomes

The ICU stay was similar between both groups (Group B 2.7 ± 4.5 days and Group A 2.2 ± 4.6 days). Overall, 66.7% of patients in Group B were discharged from the ICU on the first day after surgery.

There were no differences between the two groups in terms of hospital mortality, delirium, rate of re-revascularization, revisions necessitated by bleeding, and occurrences of stroke or atrial fibrillation, as well as postoperative pneumonia and wound infections. Moreover, the duration of hospital stay for severely obese patients (12.3 ± 9.6 days) was comparable to that of non-severely obese patients (10.8 ± 8.6 days).

In both cases requiring revision for bleeding within the obesity group, re-exploration was successfully performed through the initial thoracotomy approach, without the need for conversion to sternotomy.

The detailed postoperative adverse events and outcomes are given in Table 3.

## 4. Discussion

Despite the technical and surgical challenges posed by higher BMI, the outcomes for severely obese patients were comparable to those of non-obese patients, highlighting the adaptability of TCRAT as a surgical option. The findings of this study underscore the feasibility and reproducibility of TCRAT across all categories of patients, including those with severe obesity.

**Figure 1:**
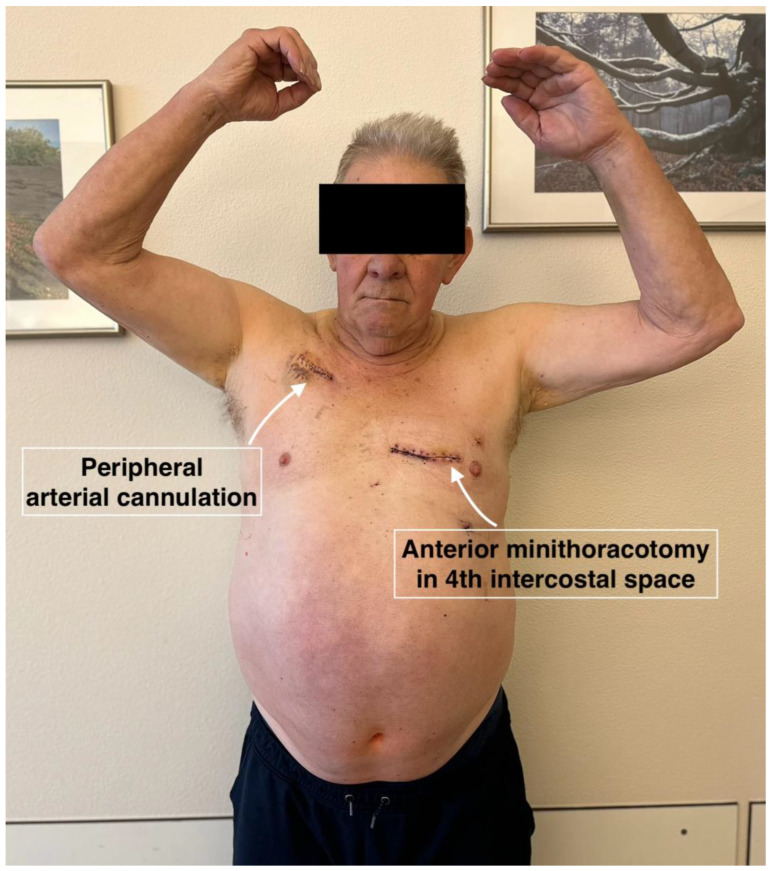
Patient on the 6th postoperative day (day of discharge) after complete arterial revascularization.

Several studies [16,17,18] have demonstrated that obese patients have a higher risk of sternal wound complications and rehospitalization. The present study shows the elimination of this issue, as the sternum remains intact. By maintaining the integrity of the sternum, complications associated with sternotomy are almost completely eliminated, leading to improved outcomes for obese patients undergoing CABG. The avoidance of sternotomy facilitated immediate patient mobilization and recovery (Figure 1). This finding emphasizes the importance of further evolution of minimal access techniques, especially in high-risk patients.

We observed a significantly longer total operation time in severely obese patients using the TCRAT technique. A major point of criticism of minimal access procedures is an increase in the complexity of the operation, which might lead to increased cross-clamp and bypass times. Those can be identified as independent predictors of morbidity and mortality after cardiac surgery [19,20]. Despite the extended operation times, the aortic cross-clamp time and CPB time were similar between groups. This finding highlights the greater complexity and technical challenge associated with surgery in severely obese patients. Likewise, the results of the present study indicate that patients with severe obesity can undergo TCRAT with outcomes similar to those of non-obese patients. The longer operation time observed in obese patients did not translate into higher rates of postoperative complications or mortality. This suggests that the technical challenges associated with operating on obese patients can be effectively managed. Previous studies on the impact of obesity on cardiac surgery outcomes have produced mixed results. Some studies have reported higher rates of complications and mortality in obese patients [19,20,21], while others have found no significant differences [18,22]. Nevertheless, this study clearly shows that TCRAT procedures can be performed within acceptable cross-clamp and CBP times even in highly obese patients. The advantage of reduced surgical trauma outweighs the adverse effects of prolonged CBP and cross-clamp times in this specific setting.

The average number of distal anastomoses performed per patient showed no significant difference between the study groups, suggesting that the degree of revascularization was unaffected by the patients’ obesity status. Complete anatomical revascularization was achieved in all patients across both groups. Building upon the existing literature, our study emphasizes the importance of meticulous perioperative care and surgical expertise to minimize the risks associated with obesity in cardiac surgeries. The absence of significant differences in postoperative complications and mortality rates between severely obese and non-severely obese patients further supports the notion that severely obese patients should not be excluded from undergoing TCRAT. The results of the present study are in line with the other studies, suggesting that tailored approaches can effectively address the unique challenges posed by obesity in surgical settings [23,24].

One finding of the present study is the lower hospital mortality rate observed in group B. This phenomenon, often referred to as the “obesity paradox”, has been reported in various cardiac surgery studies where obese patients have been observed to have similar or even better survival rates compared to their non-obese counterparts [17,25,26]. While the exact mechanisms underlying this paradox remain unclear, several hypotheses have been proposed, including the potential protective effects of higher metabolic reserves and the possible differences in inflammatory responses in obese individuals.

The comprehensive analysis presented here adds nuance to the current understanding of TCRAT outcomes, particularly in the context of severe obesity. By demonstrating comparable results across patient categories, the present study provides reassurance to clinicians considering TCRAT for obese patients, expanding the pool of candidates eligible for this minimally invasive approach. Moreover, the inclusion of various results, including infection rates and mortality, offers a holistic view of the procedure’s safety and efficacy in diverse patient populations.

In conclusion, severe obesity should not be considered as a contraindication for TCRAT. With appropriate perioperative management, TCRAT can be safely and effectively performed in obese patients, achieving complete anatomical revascularization with comparable postoperative outcomes to non-obese patients. By providing evidence-based insights, we hope to encourage broader adoption of TCRAT in diverse patient populations, ultimately improving access to minimally invasive cardiac surgery and enhancing patient outcomes. These findings support the use of TCRAT as a viable minimally invasive surgical option for complete coronary revascularization in obese patients.

### Strengths and Limitations

This study boasts several strengths, notably its large sample size and thorough examination of both intraoperative and postoperative outcomes. Nonetheless, certain limitations should be acknowledged.

First, the retrospective nature and single-center design of this study may limit generalizability, although the robust methodology and large sample size enhance the reliability of the findings. However, further multicenter prospective studies are warranted to validate these results and to explore long-term outcomes—such as graft patency, freedom from reintervention, and survival—that lie beyond the scope of this analysis. With regard to long-term results, it is expected that a higher rate of major adverse cardiovascular and cerebrovascular events will occur in the group of severely obese patients due to existing comorbidities, especially diabetes mellitus [24]. Additionally, ongoing research should focus on refining perioperative strategies to optimize outcomes for obese patients undergoing TCRAT. This includes addressing specific concerns such as the optimization of instruments and techniques, optimizing perioperative management including analgesic regimen and early recovery strategies, as well as ensuring long-term durability and efficacy of the procedures. Future investigations should be part of an integrated approach to cardiac surgery and anesthesiology, which includes comprehensive patient screening and preparation, strategic anesthetic management, advancements in minimally invasive surgical techniques, and rigorous postoperative care. This care should be provided in intensive or intermediate care units, employing specialized pain management protocols.

Moreover, the retrospective nature of this study introduces a risk of selection bias, and the single-center design may limit the applicability of the results. To validate these findings, additional multicenter prospective research is required.

Additionally, the study’s findings are constrained by a relatively short follow-up period.

## Figures and Tables

**Table 1:** Preoperative baseline characteristics before propensity score matching.

Variables	Group A (*n* = 459)	Group B (*n* = 43)	*p*-Value
Age (years)	67.5 ± 9.7 (32–88)	63.7 ± 7.3 (50.2–78)	0.006
Male, *n* (%)	400 (87.1)	36 (83.7)	0.396
BMI, kg/m^2^	27.5 ± 3.6 (18–34.9)	37.8 ± 2.2 (35.2–42.6)	<0.001
LVEF	49.1 ± 9.9 (10–65)	47.8 ± 9.8 (25–60)	0.210
EuroScore II, %	3.0 ± 2.9 (0.4–29.6)	3.3 ± 2.6 (0.6–11.0)	0.223
NYHA III, *n* (%)	386 (84.1)	36 (83.7)	0.101
CCS 3–4, *n* (%)	423 (92.2)	40 (93)	0.234
Diabetes mellitus, *n* (%)	151 (32.9)	31 (72.1)	<0.001
Arterial hypertension, *n* (%)	419 (91.3)	37 (86.1)	0.294
COPD, *n* (%)	80 (17.4)	11 (25.6)	0.080
Smoker, *n* (%)	137 (29.8)	17 (39.5)	0.088
Prior PCI, *n* (%)	113 (24.6)	9 (20.9)	0.303
Peripheral arterial disease, *n* (%)	224 (48.8)	24 (55.8)	0.151

Values are expressed as mean ± SD; minimum–maximum values are in parenthesis. BMI: body mass index, NYHA: New York Heart Association, CCS: Canadian Cardiovascular Society, COPD: chronic obstructive pulmonary disease, PCI: percutaneous coronary intervention.

**Table 2:** Operative characteristics.

Variables	Group A (*n* = 459)	Group B (*n* = 43)	*p*-Value
LIMA, *n* (%)	453 (98.7)	41 (95.3)	0.286
Radial artery, *n* (%)	300 (65.3)	30 (69.8)	0.214
Total arterial revascularization, *n* (%)	184 (40)	15 (34.9)	0.268
Multi arterial revascularization, *n* (%)	122 (26.6)	14 (32.5)	0.174
Distal anastomoses, *n*	3.0 ± 0.8 (2–5)	3.1 ± 0.7 (2–4)	0.194
Total operation time, min	320.2 ± 73.4 (145–618)	353.5 ± 83.6 (212–705)	0.003
CPB time, min	155.0 ± 42.9 (52–313)	163.3 ± 35.0 (102–275)	0.078
Aortic cross-clamp time, min	95.6 ± 33.0 (22–255)	97.9 ± 27.6 (45–148)	0.307

Data are expressed as mean ± SD; minimum–maximum values are in parenthesis. LIMA: Left Internal Mammary Artery.

**Table 3:** In-hospital outcome.

Variables	Group A (*n* = 459)	Group B (*n* = 43)	*p*-Value
Length of stay in ICU, days	2.2 ± 4.6 (1–70)	2.7 ± 4.5 (1–26)	0.225
Delirium, *n* (%)	35 (7.6)	5 (11.6)	0.167
Atrial fibrillation, *n* (%)	45 (9.8)	5 (11.6)	0.410
Pneumonia, *n* (%)	6 (1.3)	0 (0.0)	0.228
Wound complication, *n* (%)	5 (1.1)	1 (2.3)	0.233
Length of hospital stay, days	10.8 ± 8.6 (4–73)	12.3 ± 9.6 (2–44)	0.142
Revision for bleeding, *n* (%)	33 (7.2)	2 (4.6)	0.276
Stroke, *n* (%)	3 (0.6)	0 (0.0)	0.300
Re-revascularization, *n* (%)	5 (1.1)	0 (0.0)	0.248
Hospital mortality, *n* (%)	5 (1.1)	1 (2.3)	0.227

Data are expressed as mean ± SD; minimum–maximum values are in parenthesis. ICU: Intensive Care Unit.

## Data Availability

The data presented in this study are not publicly available due to ethical and privacy restrictions. Pseudonymized datasets are available from the corresponding author upon reasonable request and with approval from the local ethics committee.

## Abbreviations

BMI	body mass index
CABG	coronary artery bypass grafting
CPB	cardiopulmonary bypass
MICS	minimally invasive coronary surgery
PCI	percutaneous coronary intervention
TCRAT	Total Coronary Revascularization via left Anterior miniThoracotomy
